# Characterization of QTL and eQTL controlling early *Fusarium graminearum* infection and deoxynivalenol levels in a Wuhan 1 x Nyubai doubled haploid wheat population

**DOI:** 10.1186/s12870-019-2149-4

**Published:** 2019-12-03

**Authors:** François Fauteux, Yunli Wang, Hélène Rocheleau, Ziying Liu, Youlian Pan, George Fedak, Curt McCartney, Thérèse Ouellet

**Affiliations:** 1 Digital Technologies Research Centre, National Research Council Canada, Ottawa, Ontario Canada; 2 Ottawa Research and Development Centre, Agriculture and Agri-Food Canada, Ottawa, Ontario Canada; 3 Morden Research and Development Centre, Agriculture and Agri-Food Canada, Morden, Manitoba Canada

**Keywords:** Fusarium head blight (FHB), Quantitative trait loci (QTL), Expression quantitative trait loci (eQTL), Doubled haploid (DH), *Fusarium graminearum*, *Triticum aestivum* L.

## Abstract

**Background:**

Fusarium head blight (FHB) is a major disease of cereal crops, caused by the fungal pathogen *Fusarium graminearum* and related species. Breeding wheat for FHB resistance contributes to increase yields and grain quality and to reduce the use of fungicides. The identification of genes and markers for FHB resistance in different wheat genotypes has nevertheless proven challenging.

**Results:**

In this study, early infection by *F. graminearum* was analyzed in a doubled haploid population derived from the cross of the moderately resistant wheat genotypes Wuhan 1 and Nyubai. Three quantitative trait loci (QTL) were identified: 1AL was associated with lower deoxynivalenol content, and 4BS and 5A were associated with reduced *F. graminearum* infection at 2 days post inoculation. Early resistance alleles were inherited from Wuhan 1 for QTL 1AL and 4BS and inherited from Nyubai for the 5A QTL. *Cis* and *trans* expression QTL (eQTL) were identified using RNA-seq data from infected head samples. Hotspots for *trans* eQTL were identified in the vicinity of the 1AL and 4BS QTL peaks. Among differentially expressed genes with *cis* eQTL within the QTL support intervals, nine genes had higher expression associated with FHB early resistance, and four genes had higher expression associated with FHB early susceptibility.

**Conclusions:**

Our analysis of genotype and gene expression data of wheat infected by *F. graminearum* identified three QTL associated with FHB early resistance, and linked genes with eQTL and differential expression patterns to those QTL. These findings may have applications in breeding wheat for early resistance to FHB.

## Background

Wheat is the most important crop in the world in terms of area and Canada is one of the largest producers with 9 million hectares cultivated and 30 million tons of grain produced in 2017 [1]. Allohexaploid bread wheat (*Triticum aestivum L.*) accounts for over 95% of the global wheat production [2]. Fusarium head blight (FHB) is a major disease of wheat in Canada and other temperate areas of the world, caused predominantly by the fungus *Fusarium graminearum* Schwabe*.* Fusarium head blight results in yield losses and contamination of kernels by trichothecene mycotoxins including deoxynivalenol (DON) and derivatives [3]. Strategies to prevent the disease include the cultivation of wheat varieties resistant to FHB, crop rotations and fungicide applications [4, 5]. Achieving improved resistance to FHB is a key goal of current breeding programs in major wheat-producing countries. Several quantitative trait loci (QTL) for FHB resistance and DON reduction have been identified, including *Fhb1* on chromosome 3BS, *Fhb2* on chromosome 6BS and *Fhb5* on chromosome 5AS [6, 7]. Markers for those QTL are used to develop wheat cultivars resistant to FHB in Canada [8].

A previous study of a doubled haploid (DH) population derived from the cross Wuhan 1 x Nyubai in greenhouse and field trials identified QTL controlling FHB symptoms on chromosomes 2DL, 3BS, and 4B and QTL controlling the accumulation of DON on chromosomes 2DS and 5AS [9]. We have revisited the phenotyping and genotyping of that population by performing experiments at an earlier stage of infection in a controlled environment, analyzing gene expression profiles from infected head samples, and using a genetic map combining single nucleotide polymorphism (SNP) and single sequence repeat (SSR) markers. Our analysis identified a novel QTL on 1AL associated with DON accumulation and confirmed the 4B and 5AS QTL previously identified in the same population [9]. In addition, differentially expressed genes (DEGs) and expression QTL (eQTL) hotspots linked with reduced FHB levels were identified using RNA-seq data.

## Results

Phenotypic measures including transcript levels for the fungal genes glyceraldehyde 3-phosphate dehydrogenase (*Fg*-GAPDH) and β-tubulin (*Fg*-βTUB) and DON concentration revealed a broad range of infection levels among DH lines derived from Wuhan 1 and Nyubai (Additional file 1). There was a high correlation between the three phenotypic measures (Spearman’s rho coefficients of 0.85, 0.92 and 0.93 for *Fg*-βTUB vs. DON, *Fg*-GAPDH vs. DON and *Fg*-GAPDH vs. *Fg*-βTUB respectively). The percentages of RNA-seq reads mapping to the *F. graminearum* genome (%*Fg* reads) were also highly correlated with the above three phenotypic measures (Spearman’s rho coefficients of 0.88, 0.91 and 0.93 for %*Fg* reads vs. *Fg*-βTUB, DON and *Fg*-GAPDH respectively).

### QTL linked with reduced *F. graminearum* and DON levels

**Fig. 1:**
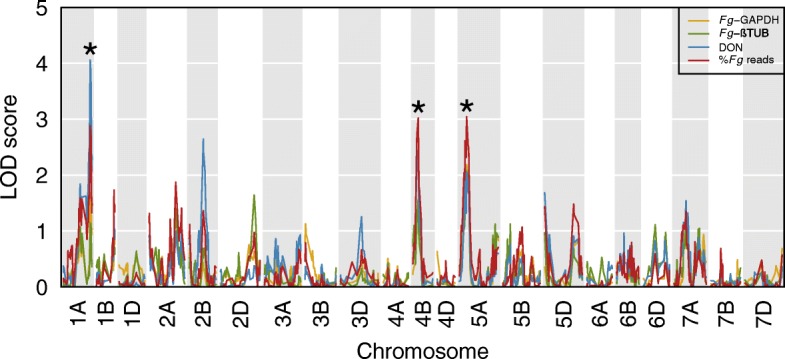
QTL LOD scores along wheat chromosomes for a set of 12,325 polymorphic markers. Stars indicate significant peaks (1AL, 4BS and 5A) above thresholds determined using 1000 permutations and *p* < =0.05

QTL mapping was performed using the level of *F. graminearum* infection at 2 dpi, as estimated by the four measures described above (%*Fg* reads, *Fg*-GAPDH, *Fg*-βTUB and DON) (Fig. 1). Three QTL were identified (*p* ≤ 0.05, 1000 permutations): a region on 1AL (peak at 151.66 cM, LOD support interval between 147.57 and 159.79 cM) was associated with DON levels; regions on chromosome 4BS (peak at 30.71 cM, LOD support interval between 15.11 and 34.72 cM) and 5A (peak at 40.50 cM, LOD support interval between 29.76 and 59.90 cM) were associated with *F. graminearum* levels (%*Fg* reads).

**Fig. 2:**
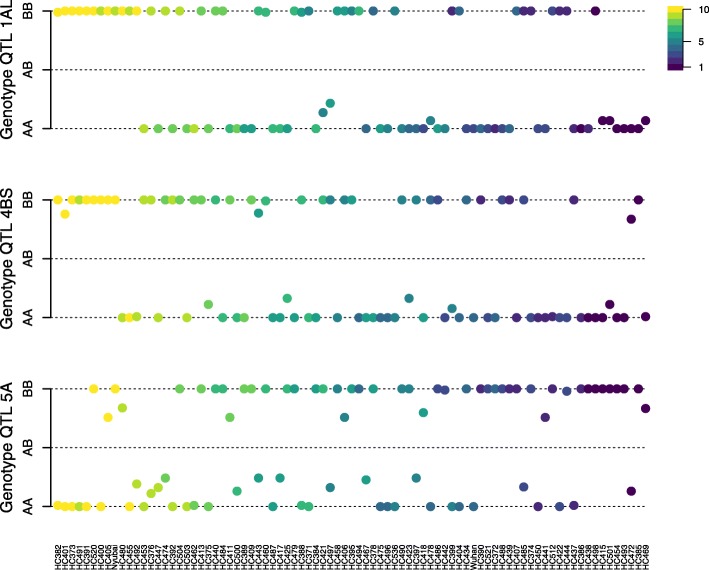
Proportion of genotype calls within 1AL, 4BS and 5A QTL regions for the two parents and 81 DH lines. Calls maximizing conditional genotype probabilities from a hidden Markov model were used to calculate proportions (vertical axis) whereas AA (Wuhan 1) and BB (Nyubai) represent 100% of the corresponding genotype within the given QTL region and AB means 50% A and 50% B. On the horizontal axis, DH lines were ordered from left to right according to the mean rank of DON concentration and %*Fg* reads. Dots corresponding to DH lines are colored according to mean of DON concentration quantiles for QTL 1AL and %*Fg* reads quantiles for QTL 4BS and 5A (yellow indicates more susceptible and dark blue more resistant). DON concentration and %Fg read values were divided into 10 quantiles (see inset)

The analysis of genotypes call frequencies within QTL 1AL, 4BS, and 5A in DH lines ordered according to phenotypes (%*Fg* reads and DON) (Fig. 2) revealed that DH lines with lower infection levels (dark blue) had genotype AA on QTL 1AL and 4BS, and genotype BB on QTL 5A, and that DH lines with higher infection levels (yellow) had the opposite pattern, which indicates that alleles associated with reduced infection levels were inherited from Wuhan 1 for QTL 1AL and 4BS and inherited from Nyubai for QTL 5A.

**Fig. 3:**
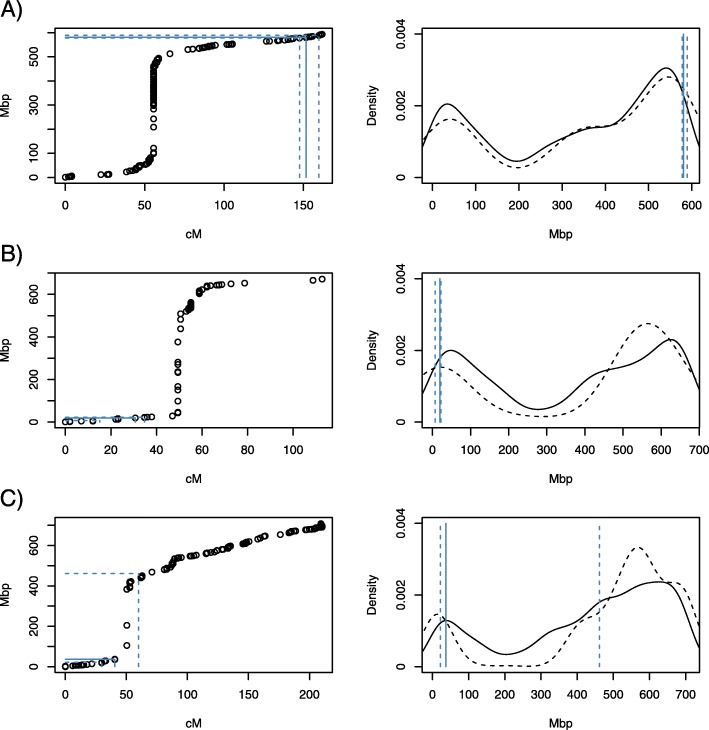
QTL peaks and LOD support intervals on chromosomes. **a** Chromosome 1A. **b** Chromosome 4B. **c** Chromosome 5A. The left panels show mapping between genetic distance (cM) and physical position (Mbp) of markers. The right panels show densities of genes (solid black line) and markers (dashed black line). Solid blue lines correspond to QTL peaks and dashed blue lines are the corresponding LOD support intervals

In order to map the physical position of genes to QTL intervals, polymorphic marker sequences were aligned against the wheat genome sequence [10] and a total of 6991 markers with consistent genetic distance and physical locations were identified (Additional file 2). Using these data, the QTL intervals were mapped to 577.89–589.41 Mbp, 7.03–22.50 Mbp and 21.67–461.45 Mbp for QTL 1AL, 4BS and 5A respectively (Fig. 3). Gene and marker densities decrease around the centromere, and regions around the centromere have lower recombination rates [11], which explains the warping between genetic distances and physical locations. The LOD support interval of 5A overlapped with the centromere, thus covering a large region of the chromosome. In total, the three QTL intervals contained 178 (1AL), 199 (4BS) and 2205 (5A) genes, respectively (Additional file 3).

### Identification of eQTL hotspots

**Fig. 4:**
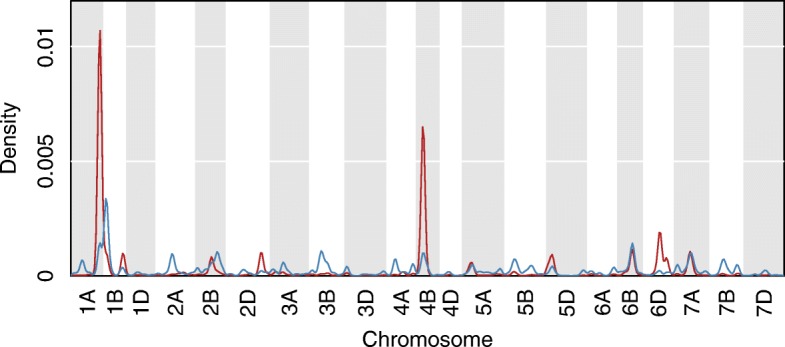
Density of *cis*-eQTL (blue) and *trans*-eQTL (red) with peaks above LOD score significance thresholds along wheat chromosomes

Expression QTL mapping was performed using RNA-seq data, and a total of 43,745 eQTL (8573 *cis* and 35,172 *trans*) with LOD score above significance thresholds (*p* ≤ 0.05, 1000 permutations) were retained, corresponding to 35,106 unique wheat genes. Among those, 240 *cis* eQTL and 17,401 *trans* eQTL were within the three QTL intervals described above (1AL, 4BS and 5A) (Additional file 4). For *cis* eQTL peaks, 54 were within the LOD support interval for QTL 1AL, 53 for QTL 4BS and 133 for QTL 5A. For *trans* eQTL peaks, 10,397 were within the LOD support interval for QTL 1AL, 6295 for QTL 4BS and 709 for QTL 5A. *Trans* eQTL hotspots were found within the 1AL (peak at 153.6 cM, interval between 144.79 and 161.42) and 4BS (peak at 30.5 cM, interval between 22.73 and 38. 27) QTL intervals (Fig. 4).

### Intersecting sets of DEGs within QTL intervals

To identify DEGs between the two parent genotypes that were also linked with observed phenotypic differences between DH lines, three analyses were performed. First, the group of DH lines with > 90% markers corresponding to Wuhan 1 (AA genotype calls) was compared with the group of DH lines with > 90% markers corresponding to Nyubai (BB genotype calls) for each QTL interval separately. A total of 4150 unique DEGs (absolute log_2_FC > =1 and adjusted *p*-value<=0.01) were identified in this analysis (3553 for QTL 1AL, 730 for QTL 4BS and 327 for QTL 5A) (Additional file 5). For the second analysis, we compared RNA-seq data (infected heads at 2 dpi) for Wuhan 1, Nyubai and one of the DH lines (HC374) between this experiment and a separate experiment [12]. These data revealed consistent differential gene expression (log2FC) between genotypes in the two experiments (Spearman’s rho coefficients of 0.90, 0.82 and 0.89 for Wuhan 1 vs. Nuybai, Wuhan 1 vs. HC374, and Nyubai vs. HC374 respectively) (Additional file 6). A total of 6452 DEGs were differentially expressed (absolute log_2_FC > =1 and adjusted *p*-value<=0.01) between the two parents (Additional file 7). For the third analysis, we compared expression data between two groups (10% of DH lines in each group) with the most extreme phenotypes ranked as described above using %*Fg* reads and DON levels: lines with the lowest infection levels (HC469, HC385, HC472, HC493, HC454, HC501, HC415, HC498) versus lines with the highest infection levels (HC382, HC401, HC373, HC491, HC391, HC520, HC400, HC405). A total of 28,254 DEGs between the two groups of DH lines were identified (absolute log_2_FC > =1 and adjusted *p*-value<=0.01) (Additional file 8). Quantitative reverse transcription-polymerase chain reaction (RT-qPCR) was used to confirm the expression profiles of five DEGs across all samples, showing correlation coefficients between 0.78 and 0.91 for RNA-seq vs. RT-qPCR (Additional file 9).

Within the three QTL intervals, a total of 13 genes were differentially expressed between genotypes, between the two parents, between the two groups of DH lines, and associated with eQTL in *cis* (eight genes in QTL 1AL, three genes in QTL 4BS and two genes in QTL 5A) (Table 1). Nine genes with higher expression in lines with lower *F. graminearum* and DON levels were considered to be associated with FHB early resistance. Among those, six genes within the 1AL QTL interval (TraesCS1A01G426000, TraesCS1A01G426500, TraesCS1A01G432900, TraesCS1A01G433000, TraesCS1A01G439000, TraesCS1A01G439100) and two genes within the 4BS QTL interval (TraesCS4B01G022400, TraesCS4B01G024600) had higher expression in genotype AA; while one gene within the 5A QTL interval (TraesCS5A01G114700) had higher expression in genotype BB. On the other hand, the four genes with higher expression in lines with higher *F. graminearum* and DON levels were considered to be associated with FHB early susceptibility. These included two genes within the 1AL QTL interval (TraesCS1A01G430100, TraesCS1A01G430200) and one gene within the 4BS QTL interval (TraesCS4B01G016900) that had higher expression in genotype BB, and one gene within the 5A QTL interval (TraesCS5A01G196700) that had higher expression in genotype AA.

**Table 1:** Differentially expressed genes between genotypes, between the two parents (Wuhan 1 and Nyubai) at 2 dpi, between two groups of DH lines (low and high infection levels) and associated with *cis* eQTL. gene_id, IWGSC RefSeq v1.0 gene ID; chr, chromosome; description, IWGSC RefSeq v1.0 gene annotation; WH, mean of normalized counts in Wuhan 1 at 2 dpi; NB, mean of normalized counts in Nyubai at 2 dpi; AA, mean of normalized counts for DH lines with genotype AA in the given QTL region; BB, mean of normalized counts for lines with genotype BB in the given QTL region; LF, mean of normalized counts for DH lines with lower levels of *F. graminearum* and DON; HF, mean of normalized counts for DH lines with higher levels of *F. graminearum* and DON; pos, position of *cis*-eQTL peak; LOD, LOD score for the *cis* eQTL peak

gene_id	chr	description	WH	NB	AA	BB	FL	FH	pos	LOD
TraesCS1A01G426000	1A	NBS-LRR disease resistance protein	89.45	0.00	72.01	0.43	53.72	0.33	151.66	45.25
TraesCS1A01G426500	1A	Agenet and BAH domain-containing protein	159.71	7.25	145.04	19.49	121.26	22.28	151.66	27.07
TraesCS1A01G430100	1A	ATP-dependent zinc metalloprotease FtsH	29.94	154.79	38.29	206.08	24.95	310.49	155.70	21.68
TraesCS1A01G430200	1A	ATP-dependent zinc metalloprotease FtsH	19.82	691.10	61.90	933.26	86.32	1425.46	155.70	31.77
TraesCS1A01G432900	1A	Na-translocating NADH-quinone reductase	74.10	0.00	64.93	0.02	56.11	0.00	154.67	51.06
TraesCS1A01G433000	1A	RNA binding protein	1371.50	185.31	548.65	132.63	568.45	99.46	154.67	31.19
TraesCS1A01G439000	1A	3-ketoacyl-CoA synthase	14.89	0.00	18.98	0.00	7.21	0.00	159.79	41.66
TraesCS1A01G439100	1A	piezo-type mechanosensitive ion channel	75.90	0.33	59.40	0.03	25.04	0.00	159.79	51.35
TraesCS4B01G016900	4B	Retrovirus-related Pol polyprotein	17.68	97.27	1.37	102.46	0.69	239.35	22.15	28.33
TraesCS4B01G022400	4B	DUF21 domain-containing protein	72.83	25.85	57.48	22.62	55.05	18.17	23.15	24.75
TraesCS4B01G024600	4B	Leucine-rich repeat protein kinase	19.07	0.55	17.20	2.25	23.16	2.31	30.71	23.16
TraesCS5A01G114700	5A	NAD-dependent protein deacylase	10.87	64.93	14.29	78.50	77.87	21.67	50.38	35.88
TraesCS5A01G196700	5A	Ubiquitin	27.43	104.77	193.48	88.74	16.33	351.23	51.59	3.45

## Discussion

Numerous QTL for FHB resistance have been identified in wheat and are distributed over all chromosomes [6, 13, 14]. The best characterized QTL for type II resistance (fungal spread within spikes) is *Fhb1*, located on the short arm of chromosome 3B [15]. Two candidate determinants of the *Fhb1* locus have been proposed: a pore-forming toxin-like gene [16] and a variant of a nuclear histidine-rich calcium-binding protein [17, 18]. In this study, we analyzed RNA-seq and genotype data in two genotypes (Wuhan 1 and Nyubai) with moderate resistance to *F. graminearum* and 81 DH lines derived from the two parents. Samples were collected at 2 dpi, which has been described as a cleavage in the host-pathogen interaction where the fungus switches from biotrophic to necrotrophic and starts producing higher amounts of DON, while the plant responds by massive transcriptional reprogramming [19]. We have identified three QTL of interest, namely 1AL at 578–589 Mbp (DON QTL and eQTL hotspot), 4BS at 7–23 Mbp (%*Fg* QTL and eQTL hotspot) and 5A at 22–461 Mbp (%*Fg* QTL). QTL located in the same regions of chromosomes 4B and 5A have been observed in a previous study with the same population at a later stage of infection [9]. QTL for FHB resistance were also reported in other populations for 1AL (*wPt-5577*-*Xbarc213*) [20], 4BS (*Xhbg226*-*Xgwm149*) [21] and 5A (*Xgwm304*-*Xgwm415*) [7]; recent work separated the latter into a major QTL mapping across the centromere and a minor QTL located on the short arm of chromosome 5A [22, 23]. QTL on 4BS (*Fhb4*) and 5AS (*Fhb5*) have both been associated with type I resistance (initial penetration) [24, 25]. As for the vast majority of FHB resistance QTL, genes underlying those QTL remain to be identified and characterized.

The strongest candidate for QTL 1AL is TraesCS1A01G426000, coding for a nucleotide-binding site leucine-rich repeat (NBS-LRR) protein; this gene had a strong differential expression between the two genotypes, higher expression in the most resistant lines, and has a regulatory function that can explain the presence of the *trans* eQTL peak detected at the same location. Most NBS-LRR proteins are cytoplasmic receptors that recognize specific pathogen molecules, triggering signaling cascades that lead to plant defense responses [26]. Several wheat NBS-LRR genes contributing to disease resistance have been mapped and are being used for improvement in breeding programs [27]. Upregulation of NBS-LRR containing genes during *F. graminearum* infection has also been reported in wheat genotypes [12, 28]. Other DEGs in the 1AL QTL interval with a *cis* eQTL close to the 1AL *trans* eQTL hotspot included an agenet and bromo-adjacent homology (BAH) domain-containing protein (TraesCS1A01G426500), a Na-translocating NADH-quinone reductase subunit (TraesCS1A01G432900) and a RNA-binding protein (TraesCS1A01G433000). Agenet and BAH domains have both been associated with recognition of epigenetic marks on histones, chromatin remodeling and regulation of gene expression [29]. Very few agenet/BAH domain containing proteins have been characterized in plants; however *Arabidopsis* EML1, an agenet-containing protein, is required for downy mildew race-specific immunity and basal defense [30]. The enzyme NADH-quinone reductase is involved in the metabolism of reactive oxygen species (ROS) and may play a role in plant defense knowing that an oxidative burst at the site of penetration is a common response in plants infected by fungal pathogens, including wheat [31]. The RNA-binding protein contains a hyaluronan-binding domain (HABP4) which indicates interactions with components of the cellular matrix, known to elicit plant defense responses [32]. Two ATP-dependent zinc metalloprotease FtsH located within the 1AL interval had higher expression in susceptible lines and were also differentially expressed between the two parents. These genes play a role in the turnover of photosystem II protein D1 and have also been associated with ROS and plant defense [33].

A single gene within the 4BS QTL had high differential expression across all comparisons and a *cis* eQTL peak located close to the 4BS *trans* eQTL hotspot. This gene (TraesCS4B01G024600) codes for an F-box/LRR-domain containing protein, a class associated with the degradation of proteins and hormone signalling [34]; this gene is a good candidate for the 4BS QTL and the data presented here points to a regulatory role for this gene in the early wheat response to FHB. Another DEG between genotypes and between most susceptible and resistant lines (TraesCS4B01G022400) codes for a DUF21 domain-containing protein, a domain found in metal transporter proteins; however the *cis* eQTL peak corresponding to this gene was relatively distant from the 4BS *trans* eQTL hotspot. Only one gene (TraesCS4B01G016900) in the 4BS QTL had higher expression associated with early susceptibility, coding for a retrovirus-related Pol polyprotein; in this case also, the *cis* eQTL was distant from the *trans* eQTL hotspot thus less likely to play a major regulatory function in the wheat response to FHB.

Our analysis did not identify a *trans* eQTL hotspot associated with the 5A QTL. Two genes in this region were differentially expressed between genotypes, between most resistant and susceptible DH lines, and associated with a *cis* eQTL. The first one, a NAD-dependent protein deacylase (TraesCS5A01G114700), had higher expression in the most resistant lines; this gene is homologous to *Arabidopsis thaliana* sirtuin 2 (SIRT2), which has been associated with mitochondrial energy metabolism and negative regulation of plant basal defense responses [35, 36]. The second gene (TraesCS5A01G196700), associated with susceptibility, codes for an ubiquitin. Ubiquitination controls different cell processes in plants including the regulation of plant defense responses [37]. A recent study by Steiner et al. [23] associated the 5A QTL with type I resistance and anther extrusion. Although included in a list of genes covered by a 5A QTL controlling anther extrusion in wheat [38], it remains unclear whether DEGs presented here play a role in this phenomenon.

Altogether, results show that combining QTL, eQTL and differential gene expression analysis enhanced the identification of candidate genes controlling early resistance to FHB in wheat. Further work is needed to confirm candidates, and may include increasing the density of markers in regions of interest, additional crosses to obtain lines carrying single QTL to facilitate downstream analyses, and genetic manipulations (e.g. genome editing [39]) to evaluate the role of each individual gene.

## Conclusion

We have identified three significant QTL: 1AL was associated with reduced DON levels, and 4BS and 5A were associated with reduced *F. graminearum* levels at 2 dpi. The resistance alleles were inherited from Wuhan 1 for the 1AL and 4BS QTL and inherited from Nyubai for the 5A QTL. *Trans* eQTL hotspots were identified at approximately the same location as QTL on the 1AL and 4BS chromosome arms. Candidate genes corresponding to QTL and eQTL were identified, including a NBS-LRR disease resistance protein in QTL 1AL and an F-box/LRR protein in QTL 4BS.

## Methods

### Plant material

Eighty-one doubled haploid lines derived from the cross Wuhan 1 x Nyubai [9, 40] were used in this study. Wuhan 1 seeds were obtained from the International Maize and Wheat Improvement Center (CIMMYT), Mexico (accession BW11778) and Nyubai seeds were obtained from the National Small Grain Collection, USA (accession PI 382154). Plants were grown in controlled-environment cabinets with 16 h light at 20 °C and 8 h dark at 16 °C until mid-anthesis. Heads at mid-anthesis were point-inoculated with 10 μL of a *F. graminearum* (strain DAOM233423, Collection of Fungal Cultures, Agriculture and Agri-Food Canada, Ottawa, Canada) macroconidial spore suspension at 1 × 10^5^ macroconidia/mL using a micropipette between the lemma and palea of two basal florets of each fully developed spikelet, on each treated head. Following inoculation, plants were transferred into a growth chamber at 25 °C where they were misted overhead; pots were disposed in a random order. Misting was for 2 days, 30 s every 1 h, during the light period. Inoculated heads were collected at 2 dpi and 5 to 6 whole heads were pooled into one sample per DH line. An aliquot of ground tissue from each sample was freeze-dried and weighed prior to quantification of the mycotoxin DON. DON analysis was performed using a DON-specific antibody and ELISA analysis [41]. The reported DON concentrations correspond to the average of two technical replicates per sample.

### RNA extraction, sequencing and reverse transcription quantitative PCR

Total RNA was extracted and processed for deep paired-end RNA sequencing using Illumina HiSeq 2500 as described in [12]. Raw data were deposited in NCBI Gene Expression Omnibus under accession GSE123548. Synthesis of cDNA and reverse transcription quantitative PCR (RT-qPCR) were performed as described in [42]. Four wheat genes were used to normalize data: glyceraldehyde-3-phosphate dehydrogenase (GAPDH, TraesCS7A01G313100), indole-3 acetaldehyde oxidase (IAAOx, TraesCS2A01G246300), amine oxidase (AOx, TraesCS2A01G327600) and heterogeneous nuclear ribonucleoprotein Q (hn-RNP-Q, TraesCS2A01G390200). For fungal biomass estimation, the expression of two *F. graminearum* genes was measured: β-tubulin (*FGSG_09530*) and GAPDH (*FGSG_06257*). Previous work showed that the expression of those two genes was highly correlated with the amount of *F. graminearum* DNA in infected plant samples and was a good estimator of fungal biomass [43]. All Primers (Additional file 10), including genome-specific primers for the genes on chromosomes 1A and 5A, were designed and synthesized as described in [42].

### RNA-seq data analysis

The International Wheat Genome Sequencing Consortium (IWGSC) RefSeq v1.0 wheat genome [44] and Ensembl Fungi (release 35) *F. graminearum* strain PH-1 genome [45] were used for RNA-seq data analysis. Wheat and *F. graminearum* genomes and gene annotations were combined prior to read alignment. RNA-seq reads were preprocessed as described in [12]. Differential gene expression analysis was performed using the DESeq2 R package [46], using the default negative binomial GLM fitting and Wald statistics. Differentially expressed genes were selected using absolute log_2_FC > =1 and Benjamini-Hochberg adjusted *p*-value<=0.01. The same procedure and parameters were used for the analysis of a different dataset [12] to identify DEGs between the two parents.

### Genotyping

Genotyping of 81 DH lines was performed using the Illumina wheat 90 K Infinium iSelect SNP array [47] and combined with previous genotyping using SSR markers [9, 48] into a revised genetic map (Additional file 11). A total of 12,325 SNPs and SSR markers were retained for the construction of the genetic map. Bins of co-segregating markers were identified using MSTmap [49]. Linkage groups were created using a minimum LOD score of 4 and maximum recombination fraction (RF) of 0.25, and recombination fractions were converted into centimorgan (cM) map distances using the Kosambi mapping function.

### Genotype data analysis

For QTL analysis, genotype probabilities and a genome scan by Haley-Knott regression were performed using R/qtl2 [50], with four phenotypic measures (%*Fg* reads, DON concentration, *Fg*-GAPDH and *Fg*-βTUB). Missing genotype data were imputed using conditional probabilities calculated using a hidden Markov model [50]. LOD support intervals were determined using a drop value of one [51]. LOD scores for eQTL were calculated using 12,325 markers and gene expression data for the 110,790 wheat genes. For this analysis, eQTL were considered *cis* if corresponding genes were on the same chromosome and within the LOD support interval, and *trans* otherwise. The distribution of eQTL peaks was evaluated using kernel densities [52, 53] using a chosen bandwidth of 10 cM, and hotspots intervals were defined using peak width at 75% height.

### Sequence alignment of markers

SNP and SSR primer sequences were respectively aligned to wheat pseudomolecules using BWA-MEM [54] and Bowtie2 [55]. A total of 9715 markers with only one best alignment with > = 95% identity and > =95% query length were retained. Of those, a total of 6991 markers with consistent genetic distance and physical locations were identified. Marker genetic positions (cM) were converted to physical positions (Mbp) using a cubic smoothing spline fit.

## Supplementary information

**Additional file 1. **Estimation of the fungal biomass in two parents and in 81 DH lines at 2 dpi. A) Percentage of *F. graminearum* reads in RNA-seq data. B) The mycotoxin DON measured by ELISA. C) *F. graminearum* GAPDH and D) β-tubulin RNA levels measured using RT-qPCR.

**Additional file 2.** Genetic distance versus physical location of markers best hits on wheat chromosomes. Markers with consistent behavior between genetic distance and physical location (6991 out of 9715 hits) are colored in black. The blue line corresponds to a cubic smoothing spline fit between the genetic distance and the physical position of the 6991 markers.

**Additional file 3.** Wheat genes within QTL intervals. gene_id, IWGSC RefSeq v1.0 gene ID; chr, chromosome; start, gene start position; end, gene end position; strand, gene strand; description, IWGSC RefSeq v1.0 gene annotation.

**Additional file 4. ***Cis* and *trans* eQTL with LOD score above significance thresholds. Type, *cis* or *trans*; chr, peak chromosome; pos, peak position in cM; lod, LOD score; ci_lo, one-LOD interval lower limit; ci_hi, one-LOD interval higher limit; gene_id, IWGSC RefSeq v1.0 gene ID; description, IWGSC RefSeq v1.0 gene annotation.

**Additional file 5. **Differentially expressed genes between genotypes within the limits of the three QTL intervals. gene_id, IWGSC RefSeq v1.0 gene ID; chr, chromosome; AA, mean of normalized counts for DH lines with genotype AA in the given QTL region; BB, mean of normalized counts for lines with genotype BB in the given QTL region; log2FC, log2 fold change; padj, adjusted *p*-value; description, IWGSC RefSeq v1.0 gene annotation.

**Additional file 6.** Comparison between differential expression in Wuhan 1, Nyubai and HC374 at 2 days post−inoculation in two experiments. Experiment 1, Pan et al. 2018; Experiment 2, this paper. A) Wuhan 1 vs. Nyubai; B) Wuhan 1 vs. HC374; C) Nyubai vs. HC374. The red line corresponds to a linear regression between the log2 fold change in the two experiments.

**Additional file 7. **Differentially expressed genes between Wuhan 1 and Nyubai at 2 dpi. gene_id, IWGSC RefSeq v1.0 gene ID; chr, chromosome; Wuhan 1, mean of normalized counts for Wuhan 1; Nyubai, mean of normalized counts for Nyubai; log2FC, log2 fold change; padj, adjusted *p*-value; description, IWGSC RefSeq v1.0 gene annotation.

**Additional file 8. **Differentially expressed genes between DH lines with extreme phenotypes. gene_id, IWGSC RefSeq v1.0 gene ID; chr, chromosome; LF, mean of normalized counts for DH lines with lower levels of *F. graminearum* and DON; HF, mean of normalized counts for DH lines with higher levels of *F. graminearum* and DON; log2FC, log2 fold change; padj, adjusted *p*-value; description, IWGSC RefSeq v1.0 gene annotation.

**Additional file 9.** Relative expression levels as quantified by RT-qPCR for five selected wheat genes, and correlation with RNA-seq data.

**Additional file 10.** Primers used for RT-qPCR analyses.

**Additional file 11.** Genetic map (cM) and genotype calls (A: Wuhan 1; B: Nyubai) for the DH population using 12,325 polymorphic markers (SSR markers and SNP from wheat 90 K Infinium beadchip array). chr, chromosome; cM, centimorgan.

## Data Availability

RNA-seq data generated in this study are available in the NCBI Gene Expression Omnibus under accession GSE123548. Analyzed data are available as Additional files to this article.

## Abbreviations

%*Fg* reads: Percentage of *F. graminearum* reads
BAH: Bromo-adjacent homology
cM: centimorgan(s)
DEG: Differentially expressed gene(s)
DH: Doubled haploid
DON: Deoxynivalenol
dpi: days post inoculation
eQTL: expression quantitative trait loci
FHB: Fusarium head blight
GAPDH: Glyceraldehyde 3-phosphate dehydrogenase
LOD: Logarithm of the odds
Mbp: Mega base pair(s)
NBS-LRR: Nucleotide-binding site leucine-rich repeat
QTL: Quantitative trait loci
RNA-seq: High-throughput RNA sequencing
ROS: Reactive oxygen species
RT-qPCR: Quantitative reverse transcription-polymerase chain reaction
SNP: Single nucleotide polymorphism
SSR: Single sequence repeat
βTUB: β-tubulin
