# Supplementary material for: Characterization of QTL and eQTL controlling early Fusarium graminearum infection and deoxynivalenol levels in a Wuhan 1 x Nyubai doubled haploid wheat population
Source: BMC Plant Biol. 2019 Dec 3;19:536. doi: 10.1186/s12870-019-2149-4 (PMC6892237; doi:10.1186/s12870-019-2149-4)

Additional file 1. Estimation of the fungal biomass in two parents and 81 DH lines at 2 dpi. A) Percentage of *F. graminearum* reads in RNA−seq data. B) The mycotoxin DON measured by ELISA. C) *F. graminearum* GAPDH and D) ß−tubulin RNA levels measured using RT−qPCR.

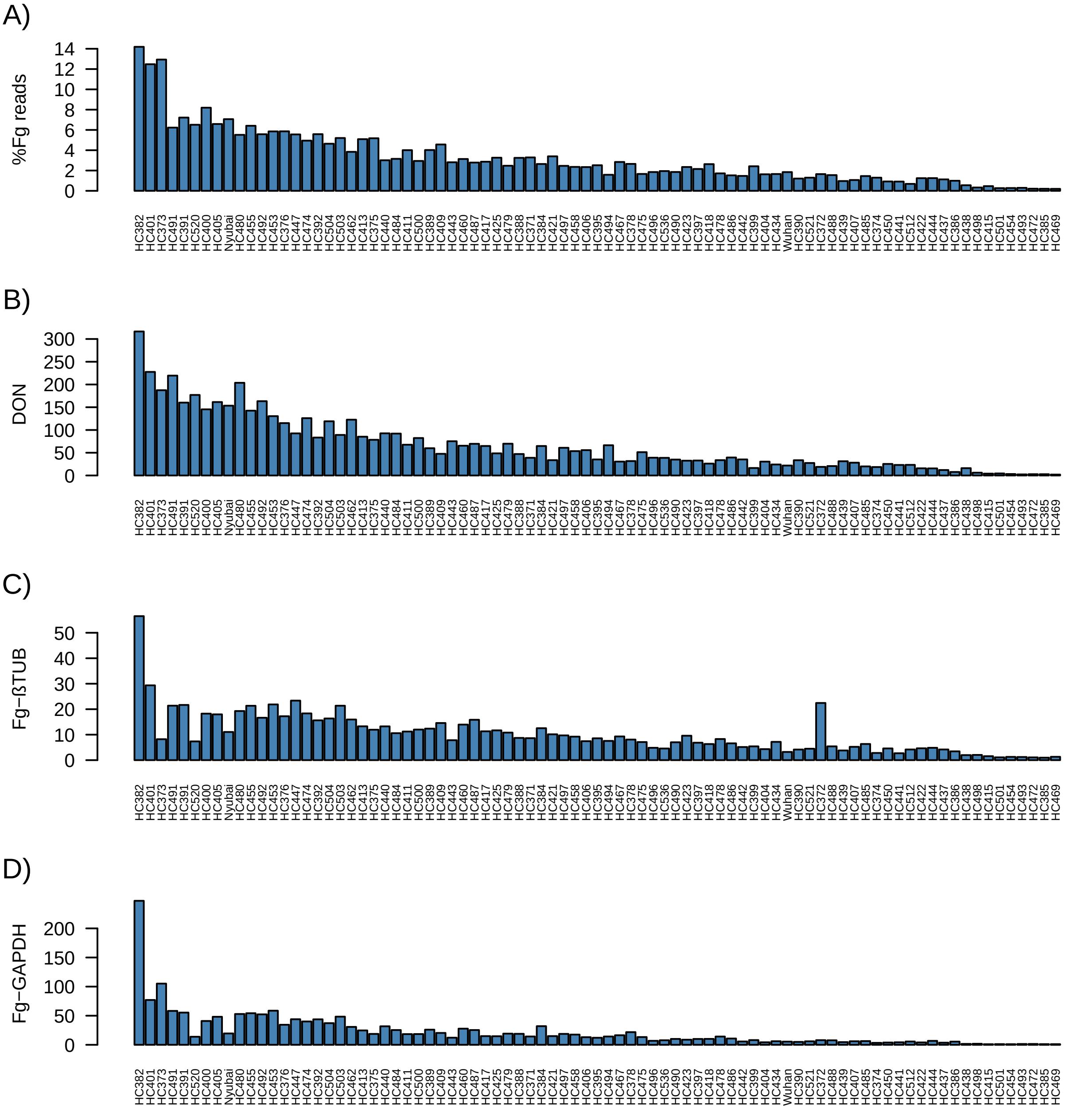

Supplement: Supplementary file 1 — Additional file 1. Estimation of the fungal biomass in two parents and in 81 DH lines at 2 dpi. A) Percentage of F. graminearum reads in RNA-seq data. B) The mycotoxin DON measured by ELISA. C) F. graminearum GAPDH and D) β-tubulin RNA levels measured using RT-qPCR. [file 12870_2019_2149_MOESM1_ESM.pdf]
