# Supplementary material for: Characterization of QTL and eQTL controlling early Fusarium graminearum infection and deoxynivalenol levels in a Wuhan 1 x Nyubai doubled haploid wheat population
Source: BMC Plant Biol. 2019 Dec 3;19:536. doi: 10.1186/s12870-019-2149-4 (PMC6892237; doi:10.1186/s12870-019-2149-4)

Additional file 2. Genetic distance versus physical location of markers best hits on wheat chromosomes. Markers with consistent behavior between genetic distance and physical location (6,991 out of 9,715 hits) are colored in black. The blue line corresponds to a cubic smoothing spline fit between the genetic distance and the physical position of the 6,991 markers.

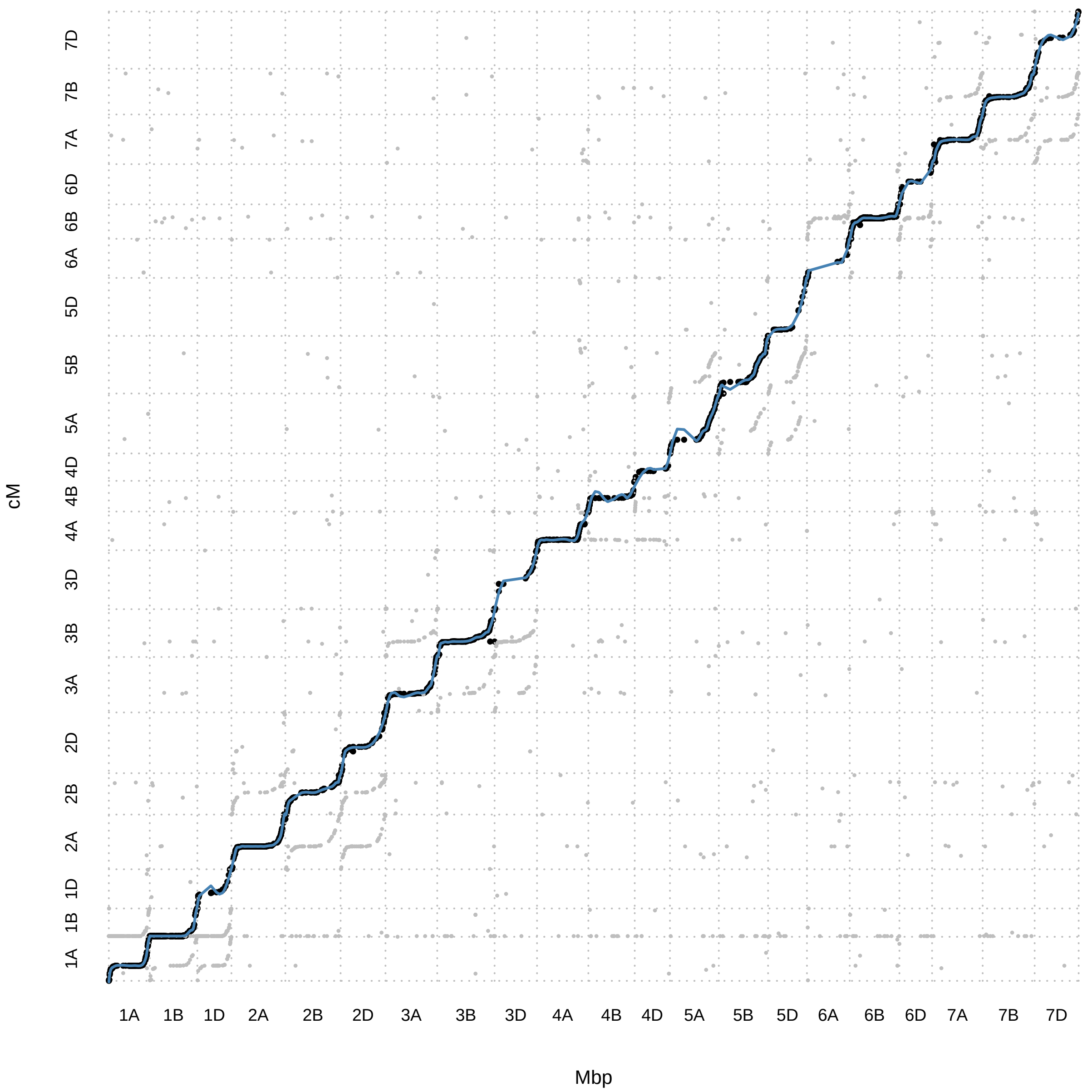

Supplement: Supplementary file 2 — Additional file 2. Genetic distance versus physical location of markers best hits on wheat chromosomes. Markers with consistent behavior between genetic distance and physical location (6991 out of 9715 hits) are colored in black. The blue line corresponds to a cubic smoothing spline fit between the genetic distance and the physical position of the 6991 markers. [file 12870_2019_2149_MOESM2_ESM.pdf]
