# Supplementary material for: Characterization of QTL and eQTL controlling early Fusarium graminearum infection and deoxynivalenol levels in a Wuhan 1 x Nyubai doubled haploid wheat population
Source: BMC Plant Biol. 2019 Dec 3;19:536. doi: 10.1186/s12870-019-2149-4 (PMC6892237; doi:10.1186/s12870-019-2149-4)

Additional file 6. Comparison between differential expression in Wuhan 1, Nyubai and HC374 at two days post−inoculation in two experiments. Experiment 1, Pan *et al.* 2018; Experiment 2, this paper. A) Wuhan 1 vs. Nyubai; B) Wuhan 1 vs. HC374; C) Nyubai vs. HC374. The red line corresponds to a linear regression between the effect size (log2FC) in the two experiments.

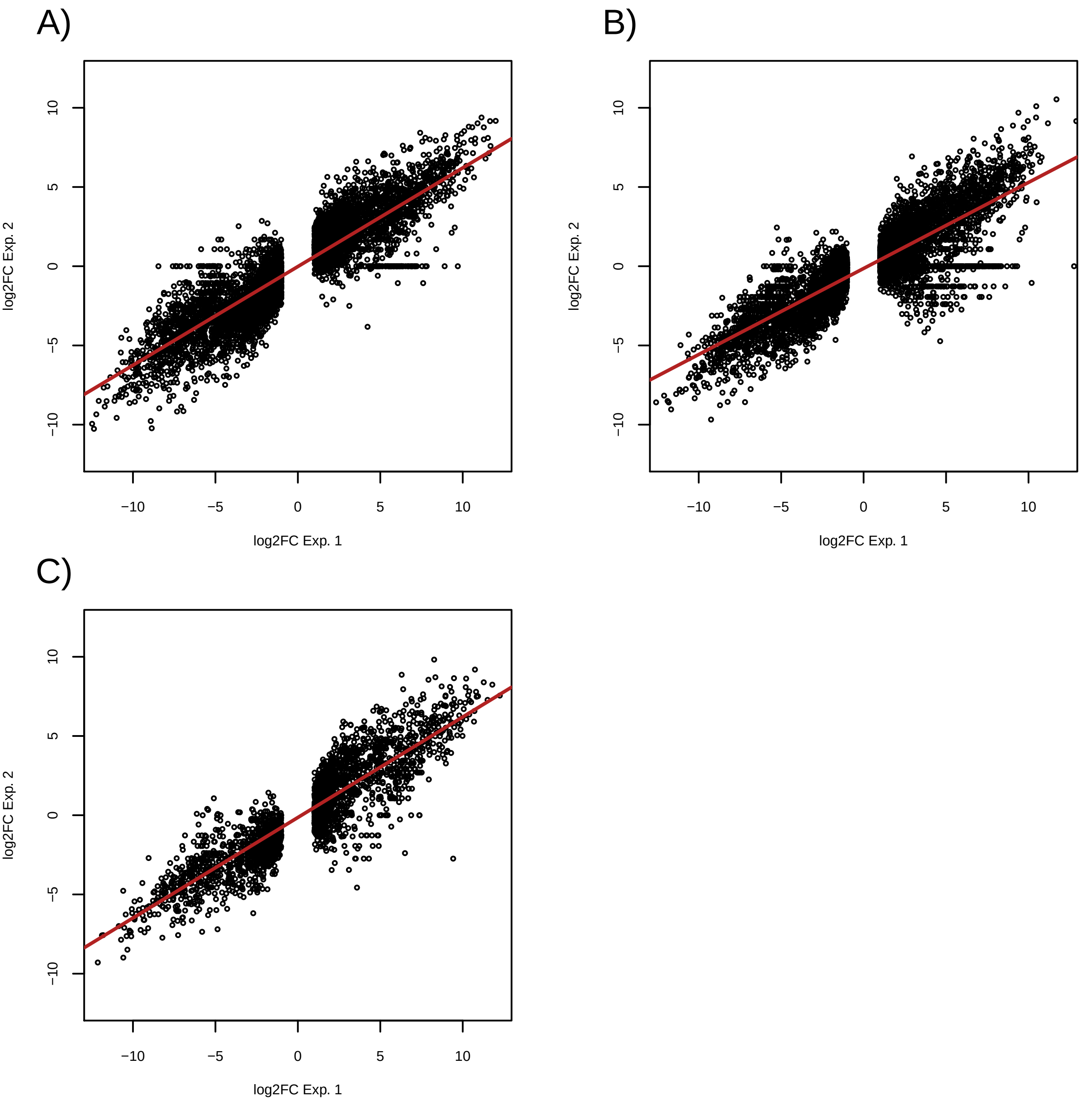

Supplement: Supplementary file 6 — Additional file 6. Comparison between differential expression in Wuhan 1, Nyubai and HC374 at 2 days post−inoculation in two experiments. Experiment 1, Pan et al. 2018; Experiment 2, this paper. A) Wuhan 1 vs. Nyubai; B) Wuhan 1 vs. HC374; C) Nyubai vs. HC374. The red line corresponds to a linear regression between the log2 fold change in the two experiments. [file 12870_2019_2149_MOESM6_ESM.pdf]
