# Supplementary material for: Characterization of QTL and eQTL controlling early Fusarium graminearum infection and deoxynivalenol levels in a Wuhan 1 x Nyubai doubled haploid wheat population
Source: BMC Plant Biol. 2019 Dec 3;19:536. doi: 10.1186/s12870-019-2149-4 (PMC6892237; doi:10.1186/s12870-019-2149-4)
Supplement: Supplementary file 10 — Additional file 10. Primers used for RT-qPCR analyses. [file 12870_2019_2149_MOESM10_ESM.docx]

**Additional file 10.** List of primers used to quantify the expression of *F. graminearum* and wheat genes by RT-qPCR.

| **Gene ID** | **Forward** | **Reverse** |  |
| --- | --- | --- | --- |
| *FgGAPDH* | TGACTTGACTGTTCGCCTCGAGAA | ATGGAGGAGTTGGTGTTGCCGTTA |  |
| *FgβTUB* | GTTGATCTCCAAGATCCGTG | CATGCAAATGTCGTAGAGGG |  |
| *TaAox* | GACTTGTCATGGTAGATGCCTG | CAGGACGAGCATAACCATTCTC |  |
| *Tahn-RNP-Q* | TCACCTTCGCCAAGCTCAGAACTA | AGTTGAACTTGCCCGAAACATGCC |  |
| *TaGAPDH* | AACTGTTCATGCCATCACTGCCAC | AGGACATACCAGTGAGCTTGCCAT |  |
| *TaIAAOx* | CACAGCAGGATTTAAGCTCTGG | GGGATGGACTAATTTCACAGGC |  |
| TraesCS1A01G430200  TraesCS1A01G435400  TraesCS1A01G424000  TraesCS5A01G120000  TraesCS5A01G080300 | AACTACCATGCCACCTACCCAGAGATT  GAGGCTGCTCTCCACACA  CGTCGTTTGCCCAGTTTC  CCTGTTTAGTCCTATTAGCCG  GGAGGACAAACTCGTTGTTGGA | CTGCTTACTTTCGATCTCAATGG  AGCTCACGGAGGAGTCTGTT  TGAGCATGCAATGCAAGTAAAATA  CTCCTGTATTATTCTTTCGGAGA  TCCAGCTCGTCATCATGGTAG |  |
|  |  |  |  |
